# Combination Antioxidant Effect of Erythropoietin and Melatonin on Renal Ischemia-Reperfusion Injury in Rats

**Published:** 2013-12

**Authors:** Nasser Ahmadiasl, Shokofeh Banaei, Alireza Alihemmati

**Affiliations:** 1 Drug Applied Research Center, Tabriz University of Medical Sciences, Medical Faculty, Tabriz, Iran; 2 Department of Physiology, Tabriz University of Medical Sciences, Tabriz, Iran; 3 Department of Histology & Embryology, Tabriz University of Medical Sciences, Medical Faculty, Tabriz, Iran

**Keywords:** Antioxidant, Erythropoietin, Ischemia Reperfusion Injury, Kidney, Lipid Peroxidation, Melatonin

## Abstract

***Objective(s):*** Renal ischemia reperfusion (IR) contributes to the development of acute renal failure (ARF). Oxygen free radicals are considered to be principal components involved in the pathophysiological tissue alterations observed during renal IR. The purpose of this study was to investigate the effect of co-administration of melatonin (MEL) and erythropoietin (EPO), potent antioxidant and anti-inflammatory agents, on IR-induced renal injury in rats.

***Materials and Methods:*** Wistar albino rats were unilaterally nephrectomized and subjected to 45 min of renal pedicle occlusion followed by 24 hr reperfusion. MEL (10 mg/kg, IP) and EPO (5000 U/kg, IP) were administered prior to ischemia. After 24 hr reperfusion, following decapitation, renal samples were taken for the determination of malondialdehyde (MDA), superoxide dismutase (SOD), catalase (CAT), glutathione peroxidase (GPx) levels and histological evaluation. The level of urea was measured in serum samples.

***Results:*** Ischemia reperfusion significantly increased urea, and MDA levels, and decreased CAT and SOD activities. Histopathological findings of the IR group confirmed that there was renal impairment in the tubular epithelium. Treatment with EPO and MEL markedly decreased urea level and increased SOD and GPx activities.

***Conclusion:*** Treatment with EPO and MEL had a beneficial effect on renal IR injury. These results may show that the co-administration of MEL and EPO cannot exert more beneficial effects than either agent alone.

## Introduction

Ischemia (cessation of blood flow), followed by reperfusion (re-establishment of blood supply), causes serious damage to tissues and organs (1, 2). Ischemia compromises the continuous supply of oxygen required by tissues to survive and maintain their physiological function. Ischemia of kidney is a common problem during kidney transplantation, partial nephrectomy, cardiopulmonary bypass, or hydronephrosis leading to renal dysfunction and injury (3- 5). Also, when reperfusion is established, additional renal reperfusion injury occurs. This involves the development of oxidative stress via generation of superoxide anions (O_2_^-^) (6). Generation of reactive oxygen species (ROS) such as O_2_^- ^and hydroxyl radical (OH) as well as reactive nitrogen species (RNS) such as nitric oxide (NO) and peroxynitrite (OONO^- ^) or the decline of antioxidant defense lead to oxidative stress, which plays a critical role in the development of renal ischemia reperfusion (IR) injury and ischemic acute renal failure (ARF) (7). 

The interaction of O_2_^-^ with NO generates OONO^-^ that causes cellular injury via DNA strand breakage and nitration of tyrosine residues on proteins (8, 9). OONO^- ^can also nitrate and deactivate antioxidant enzymes such as superoxide dismutase (SOD), contributing to further renal IR injury by promoting oxidative stress (10, 11). Enzymatic antioxidant activity involves the removal of O_2_^- ^and hydrogen peroxide (H_2_O_2_) by catalase (CAT), glutathione peroxidase (GPx), and SOD. Excessive ROS generation and decreased antioxidant defense, or both, contribute to IR injury. ROS scavengers and antioxidants that remove ROS can protect against renal IR injury (12-15). ROS generated under physiological conditions are kept under control by the action of antioxidant enzymes such as SOD, which rapidly dismutates O_2_^-^, as well as GPx, and CAT, which break down H_2_O_2_, the product of SOD, preventing the generation of damaging OH. However, during renal IR, accumulation of ROS and reductions in antioxidant enzyme expression and activities, lead to intensive damage to cellular components such as DNA, lipids and proteins. For example, endogenous SOD is rapidly depleted during renal IR, with the length of the ischemic period being the main factor (16-18). NO, OONO^-^ and ROS, cause profound injury to renal cell structures, particularly those of the proximal tubular cell. A major result is ATP depletion, which contributes to renal cell dysfunction and damage. Cell death occurs via a combination of apoptosis or necrosis, depending on the level of oxidative stress (19). Furthermore, there is interdependency between lipid peroxidation and oxidative stress. Lipid peroxidation is a catalytic mechanism leading to oxidative destruction of cellular membranes. Lipid peroxidation related to IR injury-induced tissue damage and malondialdehyde (MDA) is an indicator of the rate of lipid peroxidation (20).

Erythropoietin (EPO) is a hypoxia-inducible hematopoietic factor, which is predominantly expressed in the kidney. It has multiple protective effects, such as antioxidant, antiapoptotic, angio-genic, and anti-inflammatory effects (21, 22). The biological effects of erythropoietin are mediated by binding to its specific cell surface receptor (EPOR), and the presence of functional EPOR in renal mesangial and tubular epithelial cells has pointed to a potential role for erythropoietin in the kidney (23, 24). One important effect of erythropoietin is reduction in apoptosis and oxidative stress (25). A recent research indicates that recombinant human EPO (rHuEPO) can provide impressive protection against IR injury of several tissues and organs including the brain, heart, liver, and lungs (26- 29). It is also revealed that renal EPO level was lowered after renal ischemia reperfusion (30).

Melatonin (N-acetyl-5-methoxytryptamine) is the major product of the pineal gland that functions as a regulator of sleep, circadian rhythm, and immune function. In addition, melatonin (MEL) has a potent ROS scavenging activity because of its capacity to act as an electron donor (31- 34). MEL and its metabolites have potent antioxidant/anti-inflammatory properties and have been proven to be highly effective in a variety of disorders linked to inflammation and oxidative stress (35- 37). MEL not only neutralizes RNS and ROS species, but also acts through stimulation of several antioxidant enzymatic systems and stabilizing cell membranes (38, 39). It activates several antioxidant enzymes including CAT, SOD, and GPx. In addition, it modulates the gene expression of several protective enzymes and reduces apoptosis and lipid peroxidation (32, 40). 

Therefore, ROS were shown to contribute to the cellular damage induced by ischemia-reperfusion. The purpose of this study was to examine the co-administration effect of EPO and MEL in the reduction of injury induced by ROS in a rat model of renal ischemia-reperfusion using both biochemical and histological parameters. 

## Materials and Methods

***Animals***

In this study, 50 male Wistar Albino rats (weighing 200–300 g) were obtained from the Experimental Animal Research Center, Medical Faculty, Tabriz University, Tabriz, Iran. The rats were housed in a room with controlled temperature (21 ± 2˚C) and humidity (60 ± 5%) in which a 12-12 hr light-dark cycle was maintained. They had free access to standard water and food. The study was approved by the University Ethics Committee.

***Surgery and experimental protocol***

Under anesthesia (75 mg/kg ketamine hydrochloride and 8 mg/kg xylazine, IP), the rat was placed at right flunk position. After minimal dissection under the last rib, right nephrectomy was performed and then, the incision was sutured. Before the ischemia, the mean arterial blood pressure (ABP) was recorded in rats using the tail-cuff method. Then, the rat was placed at left flunk position, after minimal dissection under the last rib, left renal pedicle (artery and vein) was exposed. It was occluded by placing an atraumatic microvascular clamp for 45 min to induce ischemia and then subjected to reperfusion for 24 hr.

***Animal groups***

The sham group (n=10) underwent only nephrectomy without occlusion. The other groups were as follows:

IR group                                (ischemic control, n = 10)

MEL + IR group                                                     (n = 10)

EPO + IR group                                                     (n = 10)

EPO + MEL + IR group                                        (n = 10)

MEL (10 mg/kg; IP) or vehicle (1% alcohol in saline) was administered 10 min prior to ischemia. MEL (Sigma, St. Louis, MO, USA) was dissolved in absolute ethanol and then diluted in saline to give a final alcohol concentration of 1% ethanol. EPO (Neorecormon, Roche, Mannheim, Germany) was administered as a 5000 U/kg single dose IP, 20 min before ischemia.

***Biochemical analysis***

Blood samples and left kidneys were obtained after 24 hr of reperfusion in each group. The left nephrectomy specimens were divided by a sagittal section into two halves. One part was frozen in liquid nitrogen and stored at -80˚C until assayed. 

**Table 1:** Biochemical measurements after 24 hr of reperfusion

	Sham group	IR group	MEL+IR group	EPO+IR group	EPO+MEL+IR group
Urea(mg/dl)	62.11±20.95	143.00±57.58^a^	102.37±17.91^b^	97.37±21.93^b^	115.00±22.87^e^
MDA(nmol/mg protein)	5.51±1.26	7.51±0.75^a^	6.52±0.92	6.17±1.08^b^	5.73±0.75b^e^
SOD(U/mg protein)	3.94±0.49	3.25±0.35^c^	4.32±0.19^d^	4.10±0.40^d^	4.20±0.41d^e^
GPx(U/mg protein)	0.76±0.08	0.70±0.08	0.84±0.05^d^	0.82±0.06^d^	0.83±0.08d^e^
CAT(k/mg protein)	0.23±0.07	0.11±0.06^c^	0.11±0.03	0.18±0.06	0.17±0.09^e^

MDA: malondialdehyde; SOD: superoxide dismutase; GPx: glutathione peroxidase; CAT: catalase; EPO: erythropoietin; MEL: melatonin; IR: ischemia reperfusion

a Significantly increased when compared with sham group, *P* < 0.05

b Significantly decreased when compared with IR group, *P *< 0.05.

c Significantly decreased when compared with sham group, *P* < 0.05

d Significantly increased when compared with IR group, *P* < 0.05

e Not significant when compared with EPO+IR and MEL+IR groups, *P *> 0.05

Afterwards, renal MDA levels, an end product of lipid peroxidation, GPx, CAT and SOD levels and antioxidant enzymes of these samples were measured. The blood samples were centrifuged at approximately 4000 g for 10 min. The urea level in the serum was determined to assess the renal function, using the Autoanalyser (Alcyon 300 USA).

***Malondialdehyde assessment***

MDA levels were measured using the thiobarbituric acid reactive substances (TBARS) method (41).

***Glutathione peroxidase and Superoxide dismutase assessment***

To measure cytosolic enzyme activity, the kidney samples were homogenized in 1.15% kCl solution. GPx activity was measured according to Paglia and Valentine using Randox (United Kingdom) (42). Tissue SOD was assayed by a spectrophotometric method based on the inhibition of a superoxide-induced reduced nicotinamide adenine dinucleotide (NADH) oxidation according to Paoletti *et al* (43).

***Catalase***

CAT activity was measured by the method of Aebi (44).

***Histological evaluation***

The other part of left renal tissues were fixed in 10% buffered-formalin solution, dehydrated in ascending grades of alcohol and embedded in paraffin. Sections of 5 μm were taken, stained with hematoxylin-eosin (H-E), and examined under light microscope (Olympus BH-2, Tokyo, Japan) in a blinded manner by pathologist. Renal tissues were evaluated in terms of tubular lumen dilation, tubular epithelial cell vacuolization, tubular epithelial cell degeneration, and interstitial inflammatory infiltration. Histological changes were scored on a 4-point scale: (-) none, (+) mild, (++) moderate, and (+++) severe damage.

***Statistical analysis***

All the data are presented as mean ± standard deviation (SD). Evaluation of differences between groups was performed using one-way analysis of variance (ANOVA) with SPSS 19.0 software. A *P*-value of less than 0.05 was considered statistically significant.

## Results

The effect of EPO and MEL on renal ischemia reperfusion injury was investigated in 45 min of renal ischemia followed by 24 hr reperfusion. Biochemical analysis results are outlined in Table 1 and the results of histological evaluation are shown in Table 2.

***Effects of ischemia reperfusion***

The urea level was significantly higher in the animals from IR group compared with those from sham group (*P* = 0.000). The level of MDA in the IR group was significantly higher than that of the sham group (*P* = 0.002). The level of SOD in the IR group was significantly lower than that of the sham group (*P* = 0.002). The level of GPx in the IR group was lower than that of the sham group, but the difference was statistically insignificant (*P* > 0.05). The level of CAT in the IR group was significantly lower than thatof the sham group (*P* = 0.003).

**Figure1:**
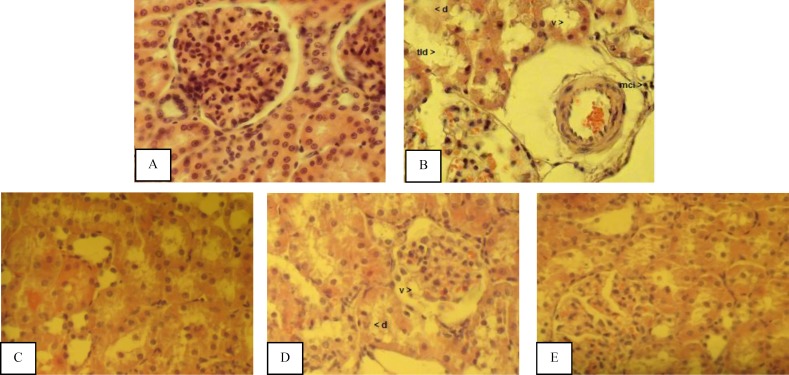
Histopathological evaluation of rat kidneys after 45 min ischemia followed by 24 hr reperfusion. Kidney sections are stained by hematoxylin and eosin (HE) and examined by a light microscope. (A) The normal renal tissue structure in the sham group. Healthy appearance of glomerular and tubular cells (40 × HE). (B) Tubular lumen dilation (tld), tubular epithelial cell vacuolization (v), tubular epithelial cell degeneration (d), and mononuclear cell infiltration (mci) in the IR group (40 × HE). (C) The normal renal tissue structure in the MEL group (40 × HE). (D) Vacuolization (v) and degeneration (d) in the EPO group (40 × HE). (E) The lower degree of vacuolization in the EPO + MEL group (40 × HE

Histological examination of the kidneys showed that there were no histological changes in the sham group (Figure 1A). In the IR group, tubular lumen dilation, vacuolization, degeneration, and mononuclear cell infiltration were higher than those of the sham group (Figure 1B).

**Table 2:** Tubulointerstitial changes in the kidney after 24 hr reperfusion (Hematoxylin and Eosin)

Groups	Tubular lumen dilation	Tubular epithelial cell vacuolization	Tubular epithelial cell degeneration	Interstitial inflammatory infiltration (mononuclear cell infiltration)
Sham	-	-	-	-
IR	+++	+++	+++	+++
MEL	+	-	+	-
EPO	++	++	++	+
EPO + MEL	+	-	+	-

A minimum of 10 fields for each kidney slide were examined and assigned for severity of changes using scores on a scale of: (–) none, (+) mild, (++) moderate, and (+++) severe damage. (n =7 for each group)

***Effects of melatonin on renal ischemia reperfusion***

Serum urea level in the MEL + IR group was significantly lower than that of the IR group (*P* < 0.05). The level of MDA in the MEL + IR group was lower than that of the IR group, but the difference was statistically insignificant (*P *> 0.05). The levels of SOD and GPx in the MEL + IR group were signi-ficantly higher than those of the IR group (*P* = 0.000). The level of CAT in the MEL + IR group was slightly lower than that of the IR group, but the difference was statistically insignificant (*P* > 0.05).

Melatonin pretreatment resulted in a marked attenuation of tubular lumen dilation, and tubular epithelial cell degeneration with absence of vacuolization and mononuclear cell infiltration induced by ischemia reperfusion (Figure 1C).

***Effects of erythropoietin on renal ischemia reperfusion***

Serum urea level in the EPO + IR group was sig-nificantly lower than that of the IR group (*P* = 0.007). The level of MDA in the EPO + IR group was significantly lower than that in the IR group (*P* < 0.05). The levels of SOD and GPx in the EPO + IR group were significantly higher than those of the IR group (*P* = 0.000). The level of CAT in the EPO + IR group was higher than that of the IR group, but the difference was statistically insignificant (*P* = 0.05). Erythropoietin pretreatment resulted in moderate tubular changes (Figure 1D).

***Effects of erythropoietin and melatonin on renal ischemia reperfusion***

In the EPO + MEL + IR group, the serum level of urea was lower than that of the IR group, but the difference was not statistically significant (*P* > 0.05). The level of MDA in the EPO + MEL + IR group was significantly lower than that of the IR group (*P* = 0.003). The levels of SOD and GPx in the EPO + MEL + IR group were significantly higher than those of the IR group (*P* = 0.000). The level of CAT in the EPO + MEL + IR group was higher than that of the IR group, but the difference was not statistically significant (*P *> 0.05). 

EPO and MEL combination treatment resulted in a marked attenuation of tubular lumen dilation and tubular epithelial cell degeneration with absence of vacuolization and mononuclear cell infiltration induced by ischemia reperfusion. Therefore, combi-nation therapy appears to have similar histological results as the melatonin treatment (Figure 1E).

## Discussion

Renal IR is a common result of clinical pro-cedures such as organ procurement, vascular surgery, or transplantation. Furthermore, renal IR injury is a leading cause of ARF, which is associated with high mortality rates. ARF is characterized by increased vascular resistance in the kidney, a low rate of filtration through the glomeruli, and tubular necrosis. These deleterious effects have been attributed to ROS generation during renal reperfusion (45, 46). The main sources of free radicals are nitric oxide synthase (NOS) and the mitochondrial electron transport chain (47, 48). ROS alter the amount of mitochondrial oxidative phosphorylation, increase intracellular calcium, deplete ATP, and activate proteases, protein kinases, and phosphatases. Thus, ROS contribute to lethal cell damage. IR injury has been attributed to ROS-mediated lipid peroxidation, which can be measured by the level of its by-products such as MDA (49, 50). 

Several experiments have revealed that renal ischemia is associated with lipid peroxidation, which is an autocatalytic mechanism causing oxidative destruction of cellular membranes, and this destruction can cause the production of reactive metabolites, toxicity and cell death (51). Lipid peroxidation, as a free radical generating system, has been proposed to be closely related to IR induced tissue injury and MDA is a good indicator of the degree of lipid peroxidation. In the present experiment, the levels of MDA are significantly increased by IR, which reflects increased lipid peroxidation due to increased oxidative stress. Erythropoietin significantly decreased the level of MDA, which shows that it decreased the amount of oxidative stress and subsequently lipid peroxidation. Consistent with our findings, Calapai *et al* (52) reported that EPO significantly decreased the level of MDA in brain tissue after cerebral IR in Mongolian gerbils. Ates *et al* (53) also demonstrated that EPO significantly decreased the level of MDA after right nephrectomy, clamping of the left renal pedicle, and reperfusion in rats. Our results show that melatonin causes a reduction in MDA production, indicating a reduction in lipid peroxidation and cellular damage. This protective effect of MEL may be in part mediated by scavenging the very reactive ONOO^- ^and OH (54). We have found that the level of MDA was significantly decreased by EPO + MEL, which indicates that EPO and MEL co-administration decreased the magnitude of oxidative stress and lipid peroxidation, but this reduction was not significant compared with the other treated groups. 

We have found that IR decreased the tissue levels of SOD, CAT, and GPx. Compatible with this finding, during IR and similar condition of oxidative stress, accumulation of ROS, reductions in antioxidant enzyme activities and expression or a combination of both cause profound injury to cellular components such as proteins, lipids and DNA. For example, endogenous SOD is rapidly depleted during renal IR, with the length of the ischemic period being a chief factor (16-18). Decreased tissue levels of SOD have been also reported in skeletal muscle damage induced by tourniquet IR method (55) and in cerebral IR damage (56). Atahan *et al* (55) proposed that overproduction of ROS during IR may cause a consumption and depletion of the endogenous antioxidant enzymes. Erythropoietin increased the tissue levels of SOD, GPx, and CAT. Consistent with our findings, Ates *et al* (53) found that EPO increased the level of glutathione (GSH) after right nephrectomy, clamping of the left renal pedicle, and reperfusion in rats. Sakanaka *et al* (57) have also reported that EPO may increase the activity of antioxidant enzymes, such as CAT, GPx, and SOD in neurons. Our results show that MEL significantly increased the levels of SOD and GPx, exhibiting the ability of melatonin dual function as both a direct reactive oxygen species scavenger and an enhancer of antioxidative enzyme activities (58). MEL stimulates several antioxidative enzymes, such as GPx and SOD, which increase its efficiency as an antioxidant (38, 59). Furthermore, the metabo-lites of MEL, such as AFMK, 6-hydroxymelatonin, and N-acetyl-5-methoxykynuramine, are documented as efficient free radical scavengers (60-62). We have found that the levels of SOD, GPx and CAT increased by EPO + MEL, which indicates that EPO and MEL combination treatment stimulates antioxidative enzymes, but this stimulation was not significant compared with the other treatment groups.

 Renal IR injury results in both glomerular and tubular dysfunctions (63). In our study, IR signi-ficantly increased urea level, suggesting an impaired glomerular function which was greatly reduced after EPO and MEL treatment. It is shown that the administration of EPO before ischemia attenuated the deterioration of renal function as a result of IR injury (53). Administration of a single dose of erythropoietin before the onset of ischemia produces a significant decrease in tubular damage, which was associated with a marked amelioration of kidney functional impairment as assessed by biochemical parameters (64). Also, it is shown that MEL affects cellular ion transportation and osmotic balance (65, 66). We explored that MEL highly improves the recovery of renal function and structure in this model, and the serum level of urea was decreased by EPO + MEL. Thus, it seems that EPO and MEL combination treatment has beneficial effects on IR-induced renal injury as indicated by lower level of urea, but this protective effect was reduced when compared with the other treatment groups. 

In our study, histological evaluation showed that IR caused changes in tubules as shown by tubular lumen dilation, vacuolization, and degeneration. Renal IR also caused an increase in interstitial inflammatory infiltration. EPO treatment attenuated the histopathological changes associated with renal IR injury. Moreover, attenuating effect of EPO on the morphological changes in renal tissue caused by IR injury has been reported (67). Sener *et al* (68) reported that melatonin has protective effects on IR-induced renal injury and the histopathological changes are reversed by MEL treatment. Also, they proposed that melatonin appears to play a cytoprotective role in the kidney insulted by ischemia reperfusion. Supporting this proposal, we have found that MEL has protective effects on tubular function. MEL severely attenuated the histopathological changes, nearly the normal renal tissue structure was preserved by melatonin pretreatment. This cytoprotective effect of MEL may be due to its powerful antioxidant properties. Also, EPO and MEL combination treatment reduced the histopathological changes in renal tissue caused by IR injury. Therefore, histological evaluation indicated that combination therapy appears to have similar histological results as the MEL treated group.

## Conclusion

In conclusion, ROS are considered to be the principal components involved in the pathophysio-logical tissue alterations observed during renal IR. Antioxidant defense systems including enzymatic antioxidant activities prevent ROS formation and scavenge ROS. The administration of EPO and MEL, which are known antioxidant agents, appears to have beneficial effects on IR-induced renal injury as indicated by lower degrees of the histopathological changes and renal dysfunction. However, combina-tion therapy cannot exert more beneficial effects than either agent alone. It seems that MEL, with its potent antioxidant properties, merits consideration as a potential therapeutic agent in renal IR injury without any need to be used as a combination therapy. However, further studies are required to clarify the exact mechanisms mediating the effect of EPO and MEL combination therapy in renal IR injury.
